# Stronger antioxidant enzyme immunoreactivity of *Populus tomentiglandulosa* extract than ascorbic acid in rat liver and kidney

**DOI:** 10.22038/ijbms.2019.34926.8296

**Published:** 2019-08

**Authors:** Choong-Hyun Lee, Joon Ha Park, Ji Hyeon Ahn, Jong Dai Kim, Jun Hwi Cho, Tae-Kyeong Lee, Moo-Ho Won

**Affiliations:** 1 Department of Pharmacy, College of Pharmacy, Dankook University, Cheonan, Chungnam 31116, Republic of Korea; 2 Department of Biomedical Science and Research Institute for Bioscience and Biotechnology, Hallym University, Chuncheon, Gangwon 24252, Republic of Korea; 3 Division of Food Biotechnology, School of Biotechnology, Kangwon National University, Chuncheon, Gangwon 24341, Republic of Korea; 4 Department of Emergency Medicine, School of Medicine, Kangwon National University, Chuncheon, Gangwon 24341, Republic of Korea; 5 Department of Neurobiology, School of Medicine, Kangwon National University, Chuncheon, Gangwon 24341, Republic of Korea

**Keywords:** Hepatic cells, Immunohistochemistry, Kidney tubules, Oxidative stress, Pathology

## Abstract

**Objective(s):**

*Populus* species have various pharmacological properties, including antioxidant activity. In this study, the effects of *Populus tomentiglandulosa* extract (PTE) on histopathology and antioxidant enzymes in the rat liver and kidney were examined.

**Materials and Methods:**

Sprague-Dawley rats were assigned to three groups; (1) normal diet fed group, (2) ascorbic acid-containing diet-fed group as a positive control, (3) PTE-containing diet-fed group. The histopathology in the rat liver and kidney was examined by hematoxylin and eosin staining. The effect of PTE was examined in the rat liver and kidney by immunohistochemistry for antioxidant enzymes, such as superoxide dismutases (SOD1 and SOD2), catalase (CAT), and glutathione peroxidase (GPx).

**Results:**

No marked histopathological alterations were observed in the liver and kidney of the PTE-containing diet-fed group. In the liver, the mean numbers of SOD1, SOD2, CAT, and GPx immunoreactive cells were significantly increased in the PTE-containing diet-fed rats, compared with those in the normal- and ascorbic acid-containing diet-fed rats. In the kidney, all SOD1, SOD2, CAT, and GPx immunoreactive structures were significantly increased in the PTE-containing diet-fed group, compared with those in the normal- and ascorbic acid-containing diet-fed groups.

**Conclusion:**

Results showed that PTE treatment significantly increased antioxidant enzymes in the rat liver and kidney, and we suggest that PTE might have hepato- and nephro-protective potentials against oxidative stress.

## Introduction

Oxidative stress occurs due to an imbalance between the production and removal of reactive oxygen species (ROS), such as hydrogen peroxide (H_2_O_2_), superoxide anions (O_2_^•^^-^), and hydroxy radicals (^•^OH). The overproduction of ROS leads to the oxidation of cellular components and cell death (1, 2). It has been reported that hepatopathy and nephropathy, which are results of hepatic and renal parenchymal injuries, are closely associated with the overproduction of ROS (3, 4). Many researchers have reported endogenous antioxidant defense mechanisms including antioxidant enzymes (superoxide dismutase [SODs], catalase [CAT], and glutathione peroxidase [GPx]) to reduce and protect against oxidative stress (5-7). For example, SODs catalyze O_2_^•^^-^ into H_2_O_2_, and CAT and GPx convert H_2_O_2_ and H_2_O, which plays coordination in the protection of oxidative damage (8). Therefore, it has been widely accepted that excessive oxidative stress is responsible for the onset and progression of cell damage/death in organs, including the liver and kidney (6, 9, 10).

The genus *Populus* (poplar) belongs to the Salicaceae family, and it has been well known that phenolic compounds and flavonoids are main components of poplar extracts, which are related to various pharmacological activities (11). In addition, many studies have reported that *Populus* species, such as *Populus nigra*, *Populus alba*, and *Populus davidiana*, display several pharmacological activities including antioxidant, anti-inflammatory, and hepato-protective (12-17). 

Although pharmacological properties of some *populous* species have been well reported, few studies on pharmacological activities of *Populus tomentiglandulosa* (Korea poplar) have been demonstrated; only our recent study has shown that *P. tomentiglandulosa* extract (PTE) has a neuroprotective effect against transient cerebral ischemia-induced hippocampal neuronal damage by attenuation of reactive gliosis (18). In addition, no studies regarding the effects of PTE on normal visceral organs have been performed yet. In the present study, therefore, we examined whether treatment with PTE represents hepatotoxicity and nephrotoxicity in normal rats, and we investigated effects of PTE on SODs (SOD1 and SOD2), CAT, and GPx, in livers and kidneys of rats.

## Materials and Methods

***Preparation of PTE***

*P. tomentiglandulosa *(specimen no.: KWNA200004212082, Kangwon National University) was collected in September 2016 in Kangwon province (South Korea) and kept in a deep freezer (-70 ^°^C). For the preparation of ethanol PTE, as described in our previous study (15), the stem and root bark of* P. tomentiglandulosa* were washed with distilled water, air-dried at 60 ± 0.5 ^°^C and ground into a fine powder by using a grinder (IKA M20, IKA, Staufen, Germany). The powder was refluxed with 70% ethanol for 24 hr at 70 ± 0.5 ^°^C. The extraction procedure was repeated three times, and the extract was filtered using Whatman No. 1 filter paper, concentrated in a vacuum evaporator and dried in a freeze-drier. The extraction yield was 14.5%. The percentage yield of the sample was calculated by comparing the weight of obtained dry extract to the initial weight of the sample.

***Experimental animals and treatment of PTE***

Male Sprague-Dawley rats (aged 12 weeks; body weight 300–320 g) were obtained from the Experimental Animal Center, Kangwon National University, Chuncheon, Republic of Korea. The rats were housed in a conventional state under adequate temperature (about 23 ^°^C) and humidity (60%) control with a 12-hr light/12-hr dark cycle. The rats were allowed free access to food and water. The procedures for animal handling and care were approved (approval no. KW-180124-1) by the Institutional Animal Care and Use Committee at Kangwon National University.

The animals were divided into 3 groups: (1) normal-group (*n*=7), which served as a negative control group and received a normal composition pellets diet; (2) ascorbic acid-containing diet-fed group (AA-group) (*n*= 7), as a positive control group; and (3) PTE-containing diet-fed group (PTE-group) (*n*=7). As described in our previous studies (19, 20), AA and PTE were mixed by 0.5% of pellets weight (w/w), respectively (Table 1). Each group was designated to receive different amounts composition of the pellets for 28 days. The age of the rats and administration period of PTE were selected based on our previous studies that showed antioxidant activities of some extracts derived from plants in the rat liver and kidney (19, 20).

***Tissue processing for histology***

For histological examination, rats in each group (*n*=7) were anesthetized with sodium pentobarbital (60 mg/kg, IP) (JW Pharmaceutical, Seoul, Korea) and perfused transcardially with 0.1 M phosphate-buffered saline (PBS) (pH 7.4) followed by 4% paraformaldehyde in 0.1 M phosphate-buffer (PB) (pH 7.4) at 28 days after AA or PTE diet. Their livers and kidneys were removed and postfixed in 10% buffered formalin. The livers and kidneys were cut sagittally, embedded with paraffin, and sectioned into 6 μm thickness on a microtome (Leica, Wetzlar, Germany).

***Hematoxylin and eosin (H-E) staining***

To examine pathological changes in the livers and kidneys of each group, H&E staining was done according to the general protocol. The sections were stained with H&E solution, dehydrated, and mounted.

***Immunohistochemistry for antioxidant enzymes***

Immunohistochemistry was carried out for SOD1, SOD2, CAT, and GPx to examine changes in antioxidants in the livers and kidneys according to our published method (19, 20). In brief, the sections were incubated with goat anti-SOD1 (1:500, Calbiochem, Darmstadt, Germany), goat anti-SOD2 (1:1000, Calbiochem), rabbit anti-CAT (1:1000, LabFrontier, Seoul, Korea), or sheep anti-GPx (1:1000, Chemicon International, Billerica, MA) as primary antibodies. Each negative control test was done by using a pre-immune serum instead of each primary antibody to establish the specificity of each immunostaining. The negative controls showed no immunoreactivity in the sections of the livers and kidneys.

For quantitatively analyzing SOD1, SOD2, CAT, and GPx immunoreactivity in the liver, digital images of the liver were taken around the central vein by using an AxioM1 light microscope (Carl Zeiss, Germany), which was equipped with a digital camera (Axiocam, Carl Zeiss, Germany) connected to a PC monitor. SOD1, SOD2, CAT, and GPx immunoreactive cells were counted at 200X magnification. Cell count in each group was obtained by averaging all numbers counted from each rat.

Ten sections of the kidney per animal were selected to quantitatively analyze SOD1, SOD2, CAT, and GPx immunoreactivity. Digital images of the kidney were captured at the renal cortex. The density of each SOD1, SOD2, CAT, and GPx-immunoreactive structure was evaluated based on optical density (OD): OD was obtained after the transformation of the mean gray level by using the formula (OD = log [256 /mean gray level]). The ratio of the OD of each immunoreactive image was calibrated as % (relative optical density, ROD) by using Adobe Photoshop version 8.0 and analyzed by using NIH Image 1.59 software (Adobe systems inc., San Jose, CA). The ratio of each ROD was calibrated as %, with the normal-group designated as 100 %.

***Statistical analysis***

All data in this study are shown as mean±SEM. A multiple-sample comparison was applied in order to test the differences between groups by ANOVA and the Tukey multiple range  *post hoc* test using the criterion of the least significant differences. Statistical significance was considered at *P*<0.05.

## Results

***H&E staining***

**Figure 1:**
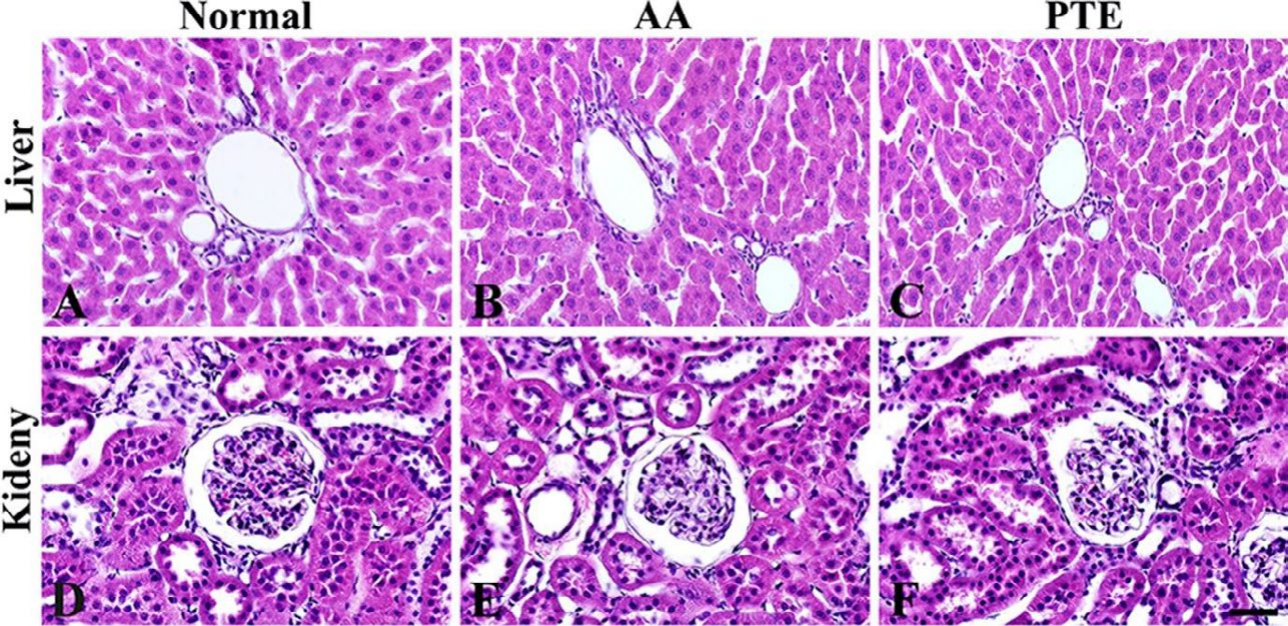
H&E staining in the liver (A-C) and Kidney (D-F) of the normal- (left column), AA- (middle column), and PTE- (right column) groups. AA and PTE administration does not affect normal hepatic and renal histology. Scale bar = 50 μm. AA: ascorbic acid; PTE: Populus tomentiglandulosa extract

Effects of PTE administration on the liver and kidney were evaluated using H&E staining (Figure 1). It was found that there were no marked histopathological alterations in the livers and kidneys of AA- and PTE-groups compared with those in the normal-group (Figure 1). These results indicate that AA and PTE did not affect normal hepatic and renal histology. 

**Figure 2:**
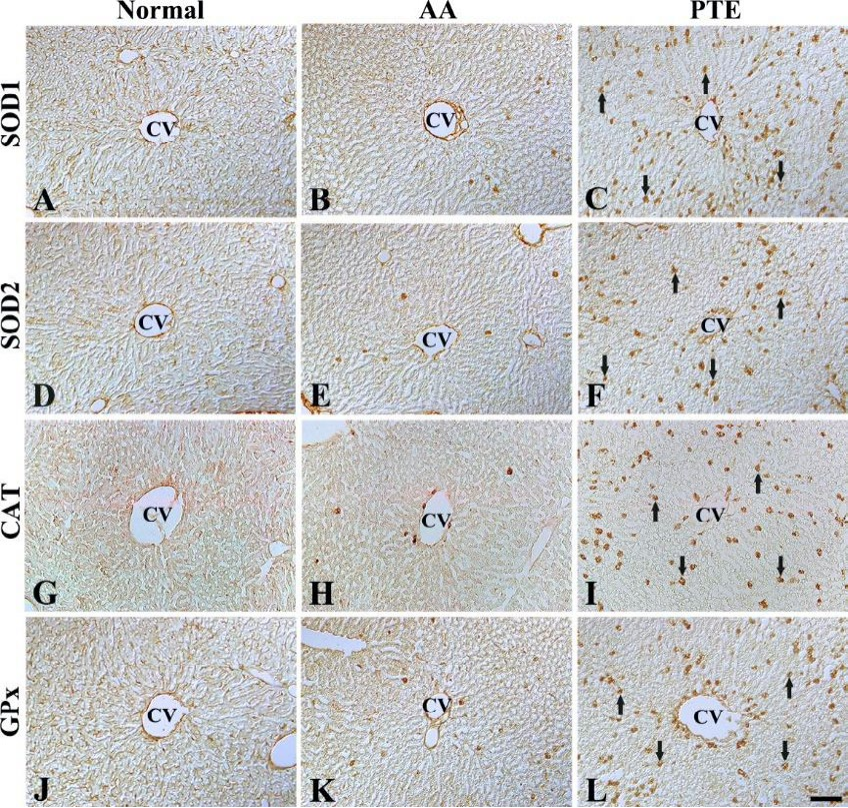
Immunohistochemistry for SOD1 (A-C), SOD2 (D-F), CAT (G-I), and GPx (J-L) in the liver of the normal- (left column), AA- (middle column), and PTE- (right column) groups. SOD1, SOD2, CAT, and GPx immunoreactive cells (arrows) are significantly increased in the PTE-group. CV, central vein. Scale bar = 200 μm. SOD1: superoxide dismutase 1; SOD2: superoxide dismutase 2; CAT: catalase; GPx: glutathione peroxidase; AA: ascorbic acid; PTE: Populus tomentiglandulosa extract

**Figure 3:**
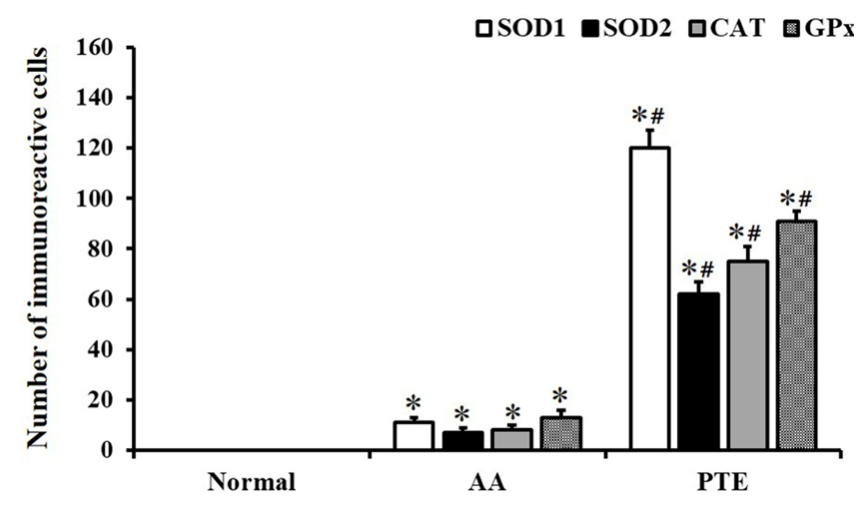
The mean number of SOD1, SOD2, CAT, and GPx immunoreactive cells in the liver of the normal-, AA-, and PTE-groups (n=7 per group; *P<0.05, significantly different from the normal-group, #*P *<0.05, significantly different from the AA-group). The bars indicate the means±SEM. SOD1: superoxide dismutase 1; SOD2: superoxide dismutase 2; CAT: catalase; GPx: glutathione peroxidase; AA: ascorbic acid; PTE: Populus tomentiglandulosa extract

**Figure 4:**
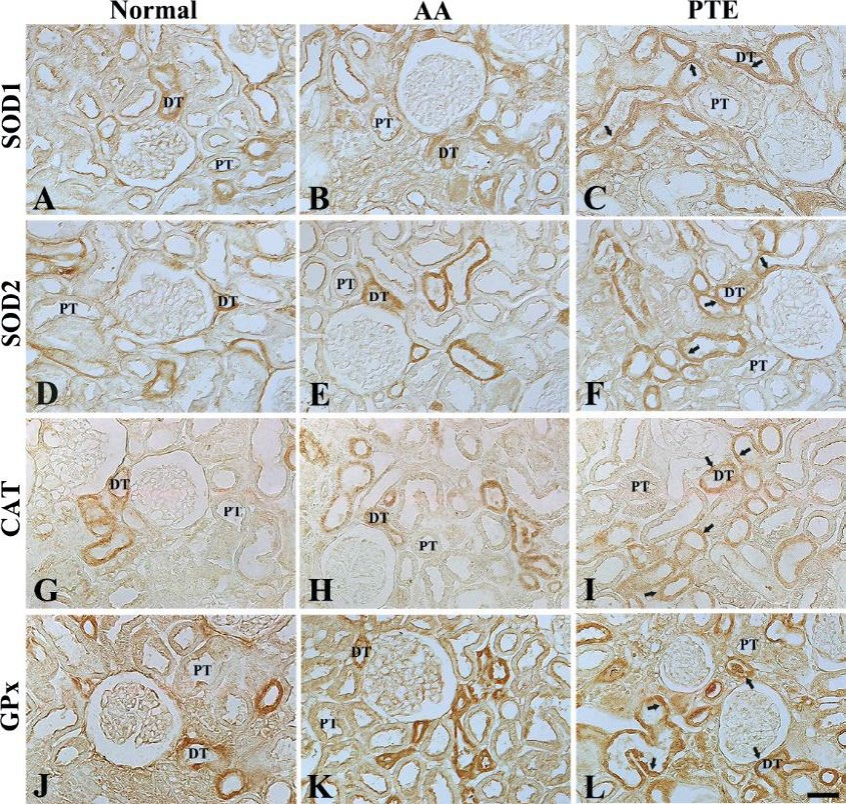
Immunohistochemistry for SOD1 (A-C), SOD2 (D-F), CAT (G-I), and GPx (J-L) in the kidneys of normal- (left column), AA- (middle column), and PTE- (right column) groups. SOD1, SOD2, CAT, and GPx immunoreactive structures are significantly increased in the PTE-group (arrows), compared to those in the normal- and AA-groups. DT, distal tubule; PT, proximal tubule. Scale bar = 200 μm. SOD1: superoxide dismutase 1; SOD2: superoxide dismutase 2; CAT: catalase; GPx: glutathione peroxidase; AA: ascorbic acid; PTE: Populus tomentiglandulosa extract

**Figure 5:**
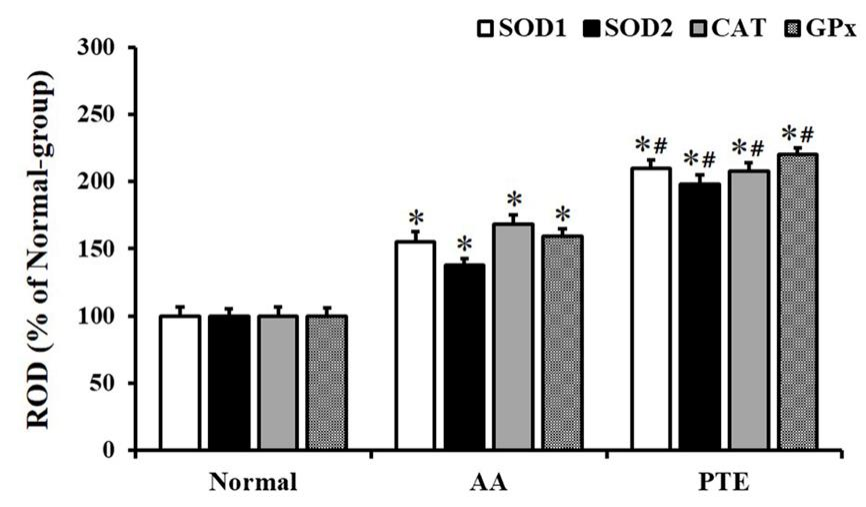
RODs of SOD1, SOD2, CAT, and GPx immunoreactive structures in the kidneys of normal-, AA- and PTE-groups (n=7 per group; *P<0.05, significantly different from the normal-group, #*P*< 0.05, significantly different from the AA-group). The bars indicate the means ± SEM. RODs: Relative optical density; SOD1: superoxide dismutase 1; SOD2: superoxide dismutase 2; CAT: catalase; GPx: glutathione peroxidase; AA: ascorbic acid; PTE: Populus tomentiglandulosa extract

**Table 1:** Composition of experimental diets

**Ingredients**	**Normal-group**	**AA-group**	**PT** **E-group**
Casein	20.0	20.0	20.0
Corn oil	5.0	5.0	5.0
Cholesterol	0.5	0.5	0.5
Corn starch	15.0	15.0	15.0
Cellulose	5.0	5.0	5.0
Mineral mix (AIN-76)	3.5	3.5	3.5
Vitamin mix (AIN-76)	1.0	1.0	1.0
Methione	0.3	0.3	0.3
Choline bitartrate	0.2	0.2	0.2
Sucrose	49.5	49	49
Ascorbic acid	-	0.5	-
*P. tomentiglandulosa* extracts	-	-	0.5

***Immunoreactivity of antioxidant enzymes in the liver***

In the normal-group, SOD1, SOD2, CAT, and GPx immunoreactive cells were hardly detected (Figures 2A, 2D, 2G, and 2J). However, a few SOD1, SOD2, CAT, and GPx immunoreactive cells were observed in the liver of the AA-group (Figures 2B, 2E, 2H, 2K, and 3). In contrast, mean numbers of SOD1, SOD2, CAT, and GPx immunoreactive cells increased significantly (*P* < 0.05) in the liver of the PTE-group compared with those in the AA-group (Figures 2C, 2F, 2I, 2L, and 3).

***Immunoreactivity of antioxidant enzymes in the kidney***

SOD1, SOD2, CAT, and GPx immunoreactivity in the kidney of the normal-group was shown in tubular cells, primarily in distal tubules (Figures 4A, 4D, 4G, and 4J). In the AA-group, all SOD1, SOD2, CAT, and GPx immunoreactivities were increased in the kidneys of the AA-group compared to those in the normal-group; each ROD of SOD1, SOD2, CAT, and GPx immunoreactive structure was about 156%, 139%, 169%, and 160% of the normal-group, respectively (Figures 4B, 4E, 4H, 4K, and 5). In addition, each ROD of SOD1, SOD2, CAT, and GPx immunoreactive structure in the kidneys of the PTE-group was more significantly increased (136%, 143%, 124%, and 138%, respectively), compared with the AA-group (Figures 4C, 4F, 4I, 4L, and 5).

## Discussion

In this study, no obvious histopathological changes were shown in the livers and kidneys of the AA- and PTE-groups, compared with the normal-group. These results indicate that PTE treatment might not lead to hepatotoxicity as well as nephrotoxicity. 

To the best of our knowledge, there is no information on antioxidant effects and compounds from *P.* *tomentiglandulosa, *but some previous studies have reported antioxidant activities of other *Populous* species. For example, Debbache-Benaida *et al*. (12) reported that ethanolic extract from *P. nigra* buds displayed antioxidant potential and attenuated hepatotoxicity induced by aluminum exposure in mice. They also reported that phenolic acids were major components of the antioxidant activity of *P. nigra*, and they urged that the hepato-protective effect of *P. nigra* buds ethanolic extract could be due to radical scavenging activity (12). Dudonne *et al*. (13) reported that treatment with *P. nigra* bud extract significantly increased catalase gene expression in both normal and aged fibroblasts from facial skin. They suggested that the potential effect of *P. nigra* bud extract on catalase gene expression reduced the detrimental effect of oxidative stress, which was related to skin aging (13). In addition, Si *et al*. (21) reported that phenolic glycosides from *P. ussuriensis* Kom. exhibited excellent antioxidant activity, suggesting that *P. ussuriensis* could be used as a good antioxidant (21).

In the present study, we examined the effect of PTE on antioxidant enzymes in the rat liver and kidney, and we found that numbers of SOD1, SOD2, CAT, and GPx immunoreactive cells were significantly increased in the liver and immunoreactivities of the 4 antioxidant enzymes were significantly increased in the kidneys of the PTE-group compared to those in the AA-group as well as the normal-group. Many researchers have reported that increased levels and activities of antioxidant enzymes, including SODs, CAT, and GPx following treatment with plant-derived extracts and their compounds display beneficial effects in livers and kidneys. In this regard, researchers (22) showed that treatment with extract from *Ocimum basilicum* leaves minimized damage induced by acetaminophen in livers and kidneys of BALB/c mice, which was closely related to the decrease of malonaldehyde (MDA, an indicator of oxidative stress) level and increase of SOD and CAT levels (22). In addition, a study (23) demonstrated that treatment with extract and active compound (6-gingerol) from *Zingiber officinale* attenuated mercuric chloride-induced hepatorenal toxicity via reducing MDA level and restoring SODs, CAT, and GPx activities in rat liver and kidney (23). Interestingly, another study (10) also reported that treatment with poplar type propolis resulted in the improvement of streptozotocin-induced liver and kidney lesions, showing that hepatorenal GPx level was increased and MDA level was decreased (10). Based on the findings, they suggested that poplar type propolis could reduce hepatorenal injury by decreased lipid peroxidation and increase of anti-oxidant enzyme activity (10). Therefore, it is likely that pretreatment with PTE might represent hepato- and nephro-protective effects via increasing antioxidant activity against hepatic and renal injury induced by oxidative stress.

## Conclusion

The data show that PTE treatment significantly increased expressions of antioxidant enzymes in the rat liver and kidney. It is suggested that PTE might have hepato- and nephro-protective potentials against oxidative stress.

## Conflicts of Interest

The authors have no financial conflicts of interest.
